# Dual size-exclusion chromatography for efficient isolation of extracellular vesicles from bone marrow derived human plasma

**DOI:** 10.1038/s41598-020-80514-8

**Published:** 2021-01-12

**Authors:** Jik-Han Jung, Woojin Back, Junyong Yoon, Hyeonjeong Han, Ka-Won Kang, Byeonghyeon Choi, Hyesun Jeong, Jaena Park, Hyunku Shin, Woojune Hur, Yeonho Choi, Sunghoi Hong, Hyun Koo Kim, Yong Park, Ji-Ho Park

**Affiliations:** 1 Department of Bio and Brain Engineering and KAIST Institute for Health Science and Technology, Korea Advanced Institute of Science and Technology (KAIST), Daejeon, Republic of Korea; 2 Division of Hematology-Oncology, Department of Internal Medicine, Korea University College of Medicine, Seoul, Republic of Korea; 3 Department of Thoracic and Cardiovascular Surgery and Department of Biomedical Sciences, Korea University College of Medicine, Seoul, Republic of Korea; 4 School of Biosystems and Biomedical Sciences, Korea University, Seoul, Republic of Korea; 5 Department of Biomedical Engineering, Korea University, Seoul, Republic of Korea

**Keywords:** Biomedical engineering, Chromatography

## Abstract

Isolation of pure extracellular vesicles (EVs), especially from blood, has been a major challenge in the field of EV research. The presence of lipoproteins and soluble proteins often hinders the isolation of high purity EVs upon utilization of conventional separation methods. To circumvent such problems, we designed a single-step dual size-exclusion chromatography (dSEC) column for effective isolation of highly pure EVs from bone marrow derived human plasma. With an aim to select appropriate column design parameters, we analyzed the physiochemical properties of the major substances in bone marrow derived plasma, which include EVs, lipoproteins, and soluble proteins. Based on these findings, we devised a novel dSEC column with two different types of porous beads sequentially stacked each other for efficient separation of EVs from other contaminants. The newly developed dSEC columns exhibited better performance in isolating highly pure EVs from AML plasma in comparison to conventional isolation methods.

## Introduction

Extracellular vesicles (EVs) are multivesicular body (MVB)-originated membranous vesicles with the heterogenous size range of 20–200 nm. They contain biomolecules such as nucleic acid, proteins, and lipids, that can provide useful information about the cells of their origin. In addition, EVs are enriched with endosome-specific tetraspanins such as CD9, CD63, and CD81^1,2^. Recently, EVs in blood have been utilized for liquid biopsies for disease monitoring and diagnosis^3–8^.

Several isolation methods including ultracentrifugation, density gradient separation, polymer-based precipitation, immunoaffinity separation, tangential-flow filtration, size-exclusion chromatography and their combinations have been utilized to purify EVs from cell culture medium or blood^9–16^. However, the presence of ApoB positive particles, soluble proteins^16^, or their aggregates often impedes the efficient isolation of highly pure EVs^12^. Although combinatorial strategies such as density gradient centrifugation and immunoaffinity based methods have been developed to separate EVs from ApoB positive particles in blood, time-consuming multiple steps lowers the yield of isolated EVs^15^. There is therefore an urgent need to develop a novel isolation technique for enriching highly pure EVs from blood.

Acute myelogenous leukemia (AML) has been chosen as the disease model in this study. Numerous studies reported the feasibility of applying plasma-derived EVs in reflecting the pathophysiology of AML^17–19^. In addition, CD63, a tetraspanin protein is found on AML associated EVs^20^. Bone marrow plasma extraction is considered as the gold standard procedure for diagnosising or monitoring AML^21^. For this reason, we conducted EV isolation from bone marrow (BM) plasma. First, every eluate fraction of conventional CL-2B columns, upon loading cell culture media (CCM) or BM plasma, was analyzed to check for the fractions with strong CD63 and ApoB signals. Note that CD63 is a well-known EV associated marker while ApoB is a protein expressed in low density lipoproteins (LDLs)/very low density lipoproteins (VLDLs). Afterwards, we performed physiochemical characterizations on the particles contained in CD63 and ApoB intensified fractions, respectively. We discovered different CD63 elution patterns for the samples of CCM and BM plasma. The eluate fractions, in which EVs are conventionally collected (after the void volume fraction), were indeed rich in ApoB positive particles.

By taking into account the physical characteristics of EVs and ApoB positive particles in plasma, we designed a dSEC column which conains two different types of porous beads sequentially stacked each other. This has been designed to separate EVs from ApoB positive particles (> 50 nm) and smaller soluble proteins (< 250 kDa), respectively. We showed that EVs were efficiently separated from other contaminants in a single isolation step by using the developed dSEC column. We further demonstrated the ability of the developed dSEC column in isolating highly pure EVs from BM plasma in comparison to conventional EV isolation methods which include polymer-based precipitation and SEC.

## Results

### CD63 purity distributions of cell culture media (CCM) and bone marrow (BM) plasma upon utilizing CL-2B columns

**Figure 1:**
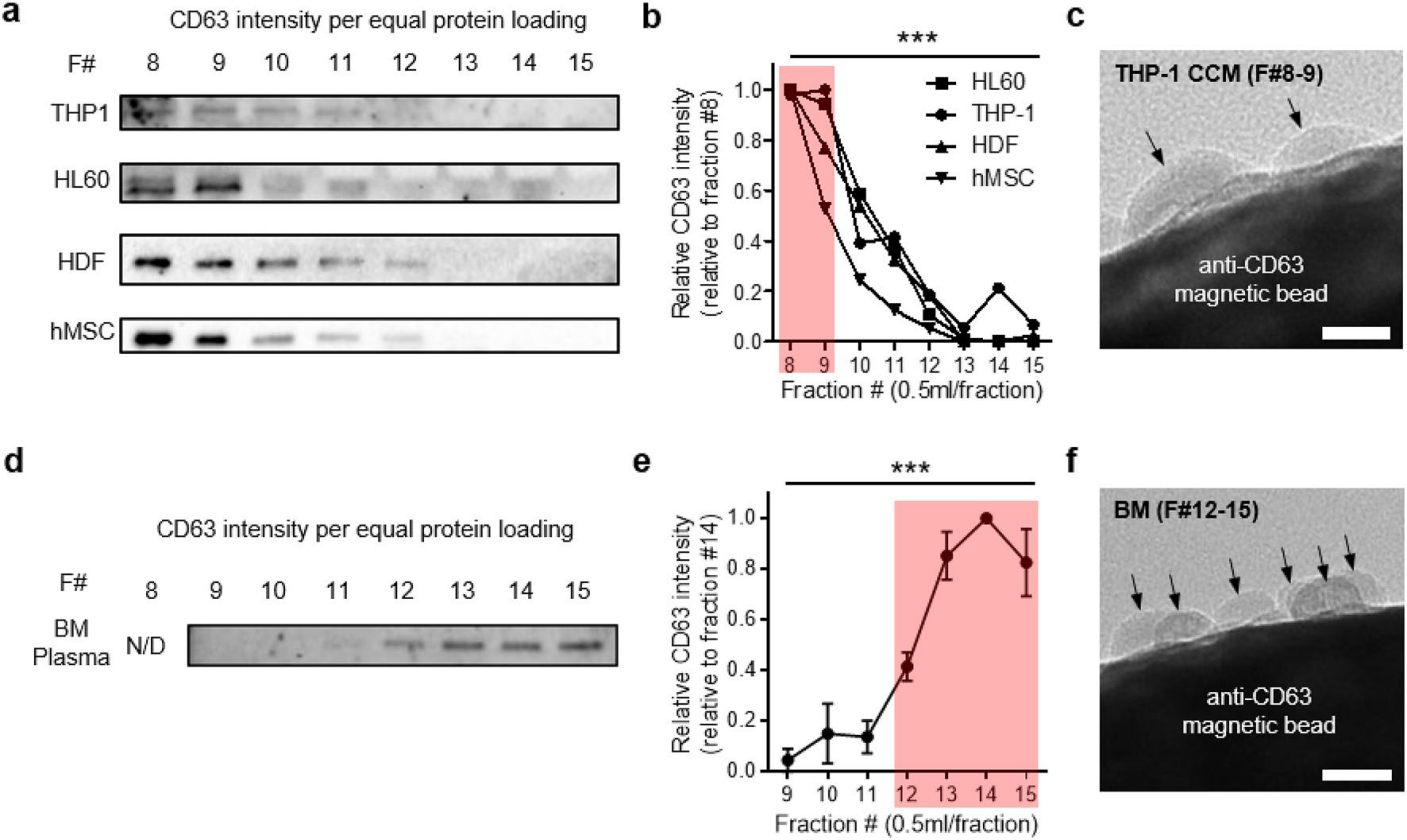
CD63 purity distributions of cell culture media (CCM) and bone marrow (BM) plasma upon utilizing CL-2B columns (**a**) Western blot analysis (equal protein loading) of CD63 on eluate fractions 8–15 of AML associated (THP-1 and HL60) and control (HDF and hMSC) cell lines. (**b**) Relative CD63 intensities of (**a**). (**c**) TEM image of anti-CD63 magnetic bead-labelled EVs collected in eluate fractions 8–9 (THP-1). EVs are marked with black arrows. Scale bar: 30 nm. (**d**) Western blot (equal protein loading) analysis of CD63 on eluate fractions 8–15 of BM plasma. N/D: Not Detectable. (**e**) Relative CD63 intensities of (**d**). (**f**) TEM image of anti-CD63 magnetic bead labelled EVs collected in eluate fractions 12–15 (BM plasma). EVs are marked with black arrows. Scale bar: 30 nm (***p < 0.001 by One-way ANOVA).

A CL-2B column, which has been widely utilized for isolating EVs^9,10,22^ was first used to enrich EVs from AML(THP-1 and HL-60) cell culture supernatants. Non-AML cells such as human dermal fibroblast (HDF) and human mesenchymal stem cells (hMSC), were utilized as controls. Western blotting performed, with equal protein loading amount, on individual eluate fractions revealed significantly higher CD63 intensities in fractions 8–9 (Fig. 1a,b, *p* < 0.001 by One-way ANOVA) when compared to eluate fractions > 10. Indeed, CD63 positive EVs were observed in these fractions (Fig. 1c). This result is in line with previously reported data which also emphasized the occurence of high CD63 intensities in early eluate fractions^10^. However, when BM plasma was used, a different trend for CD63 intensity was exhibited, even though the same CL-2B column was utilized. Unlike CCM, significantly higher CD63 intensities were noted in fractions 12–15 when compared to fractions 9–11 (Fig. 1d,e, *p* < 0.001 by One-way ANOVA). Anti-CD63 magnetic beads were then co-incubated with the particles in fractions 12–15 and visualized under TEM. We could clearly visualize CD63 positive EVs attached on the surface of magnetic beads (Fig. 1f). The EVs in fractions 12–15 were having the density of 1.214–1.318 g/ml, indicating the presence of high density EVs (1.26–1.29 g/ml)^23^ (Supplementary Fig. S1a). Furthermore, EVs in these fractions were having the zeta potential peak at—13.3 mV (Supplementary Fig. S1b). The collected EVs were having the size range of 30–200 nm when measured using NTA (Supplementary Fig. S1c).

### Enrichment of BM derived ApoB positive particles in eluate fractions 9–11 of CL-2B columns

**Figure 2:**
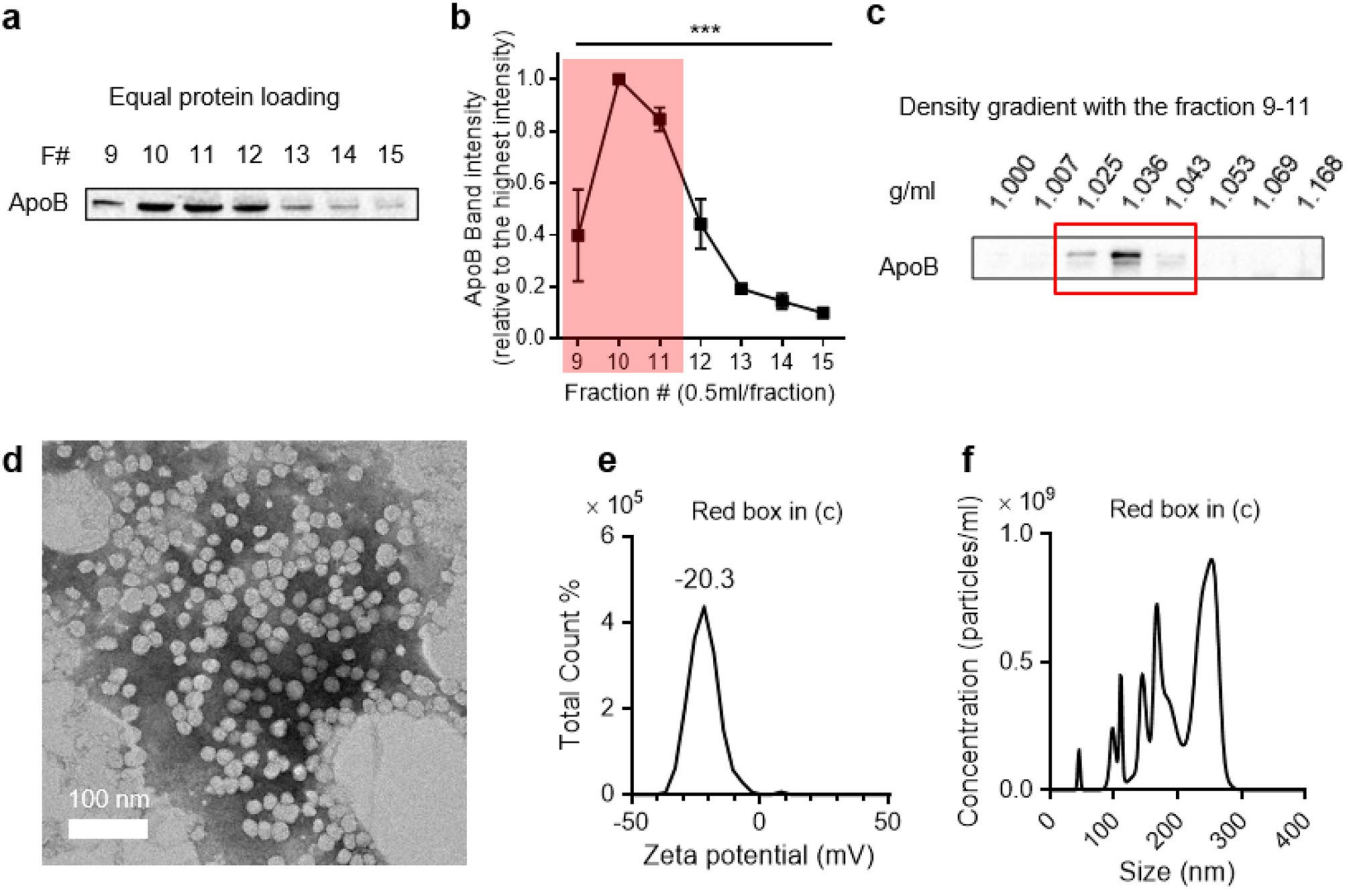
Enrichment of BM derived ApoB positive particles in eluate fractions 9–11 of CL-2B columns. (**a**) Western blot (equal protein loading) analysis of ApoB on eluate fractions 9–15 of BM plasma. (**b**) Relative ApoB intensities of (**a**). (**c**) Western blot analysis of ApoB in eluate fractions 9–11 of AML BM plasma after density gradient ultracentrifugation. The particles with the density in the range of 1.025–1.043 g/L (red box) were collected for subsequent characterizations demonstrated in Figs. 2D–F. (**d**) Visualization of the isolated ApoB positive particles under TEM. (**e**) Zeta potential of the isolated ApoB positive particles. (**f**) Size distribution of the isolated ApoB positive particles. (***p < 0.001 by One-way ANOVA).

To provide the possible explanation for the discrepancies in CD63 intensity patterns, the presence of ApoB (LDLs/VLDL marker) in each individual eluate fraction was investigated. The existence of ApoB positive particles in plasma has long been a problem for collecting high purity EVs when utilizing SEC^1,13,28,29^. Indeed, western blotting on eluate fractions of BM plasma revealed that the band intensities corresponding to ApoB, a well-known LDL/VLDL marker, was significantly enriched in fractions 9–11 compared to the fractions eluted later (Fig. 2a,b, *p* < 0.001 by One-way ANOVA). When fraction 10, which exhibited the highest ApoB intensity, was visualized under TEM, spherically shaped particles were observed (Supplementary Fig. S2). To further characterize the ApoB positive particles present, iodixanol density gradient centrifugation was conducted on fractions 9–11. The density of the ApoB positive particles present was in the range of 1.025–1.043 g/ml, similar to the density of LDLs, and not VLDLs (< 1.006 g/ml) (Fig. 2c)^24^. Furthermore, the morphology and the zeta potential (− 20.3 mV) of these particles were similar to commercial LDLs (Fig. 2d,e, and Supplementary Fig. S3). In addition, NTA determined the size of the ApoB positive particles to be in the range of 100–300 nm (Fig. 2f). These results all indicate that aggregation was occured in LDLs, and not in VLDLs which ultimately led to the enrichment of ApoB positive particles in fractions 9–11 upon SEC of BM plasma. Previous reports revealed that LDLs tend to aggregate or fuse under temperature fluctuations as in the case of freezing and thawing (F/T) in vitro^25,26^. As such, we hypothesized that the cycles of F/T on plasma would also increase the size of LDLs, possibly by inducing aggregation. We verified this phenomenon by utilizing commercial LDLs in-vitro. Upon freezing and thawing, there was an increase in the size of LDLs due to aggregation/fusion (Supplementary Fig. S4). However, this effect was not applicable to EVs and soluble proteins (Supplementary Fig. S5).

### Efficient removal of ApoB positive particles by CL-6B columns

**Figure 3:**
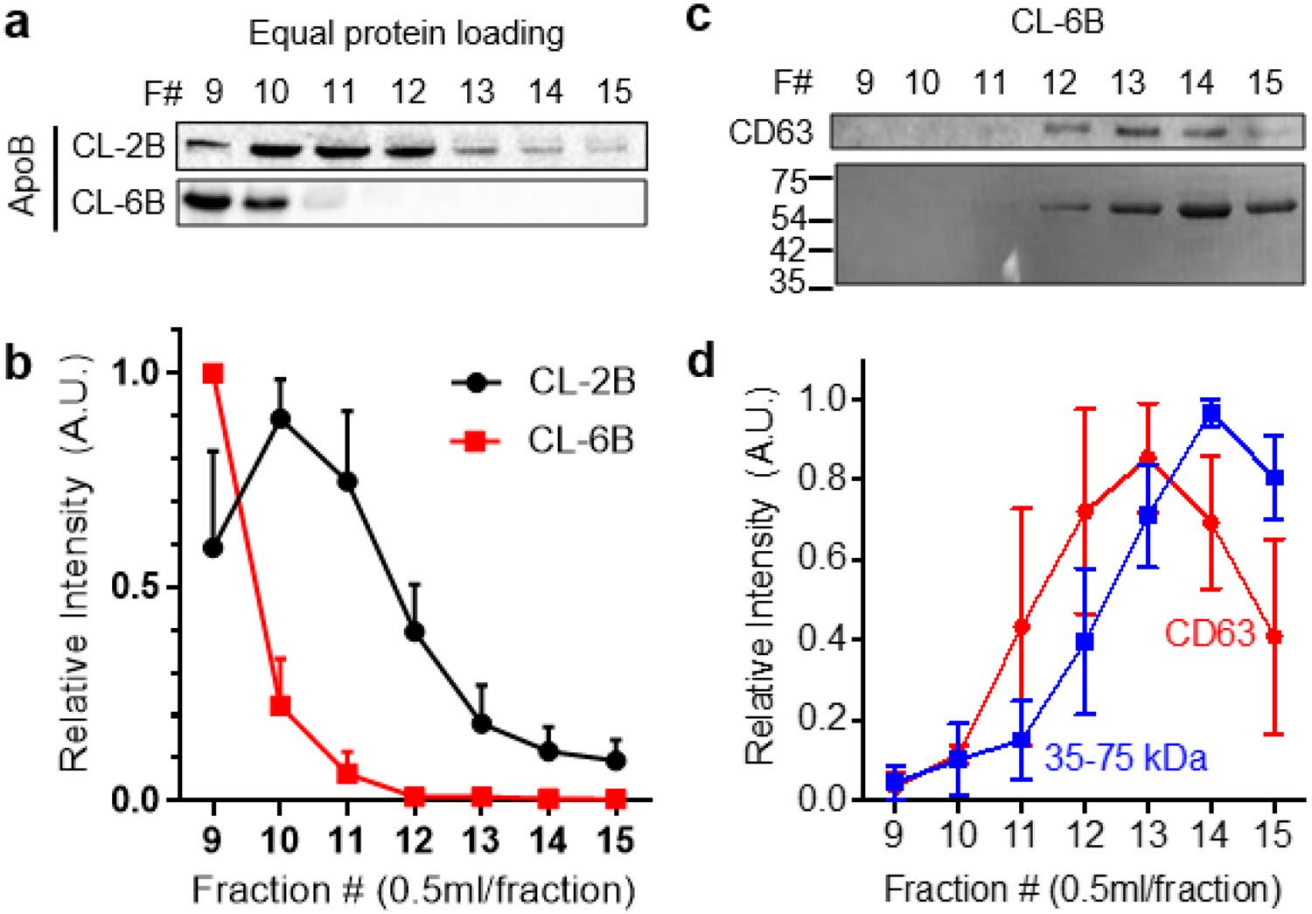
Isolation of aggregated LDLs by CL-6B columns. (**a**) Western blot (equal protein loading) analysis of ApoB on eluate fractions of BM plasma either separated by CL-2B or CL-6B columns. (**b**) Relative intensities of ApoB bands obtained in (**a**) (n = 3). (**c**) Western blot (equal protein loading) analysis of CD63 and soluble proteins (MW: 35–75 kDa) on eluate fractions of BM plasma separated using the CL-6B column. (**d**) Relative intensities of ApoB and soluble protein bands in (**c**) (n = 3).

High proportion of ApoB positive particles was detected in eluate fractions 9–11 of CL-2B columns (Fig. 2b). As such, a new SEC column was constructed with Sepharose CL-6B beads (CL-6B columns) which have the pore size in the range of 10–50 nm^27^. The pore size of CL-6B was suffcient for effectively separating EVs from the aggregated ApoB positive particles. Western blotting performed, with equal protein loading amount, on individual eluate fractions of the CL-6B column revealed the intensification of ApoB bands in fractions 9–10 unlike the CL-2B column (Fig. 3a,b). In addition, CD63 bands were detected in fractions 11–15 (Fig. 3c,d). Afterwards, in order to determine whether CL-6B columns were also effcient in isolating EVs from soluble proteins such as albumin, γ-globulin, and fibrinogen, which comprise approximately 90% of total human plasma proteins^28^, ponceau S staining and western blotting were performed post SDS-page gel electrophoresis on individual eluate fractions. The bands in 35–75 kDa range, which correspond to soluble proteins, were overlapping with CD63 bands especially in eluate fractions 12–15 (Supplementary Fig. S6).

### Efficient removal of ApoB positive particles and soluble proteins by dSEC columns

**Figure 4:**
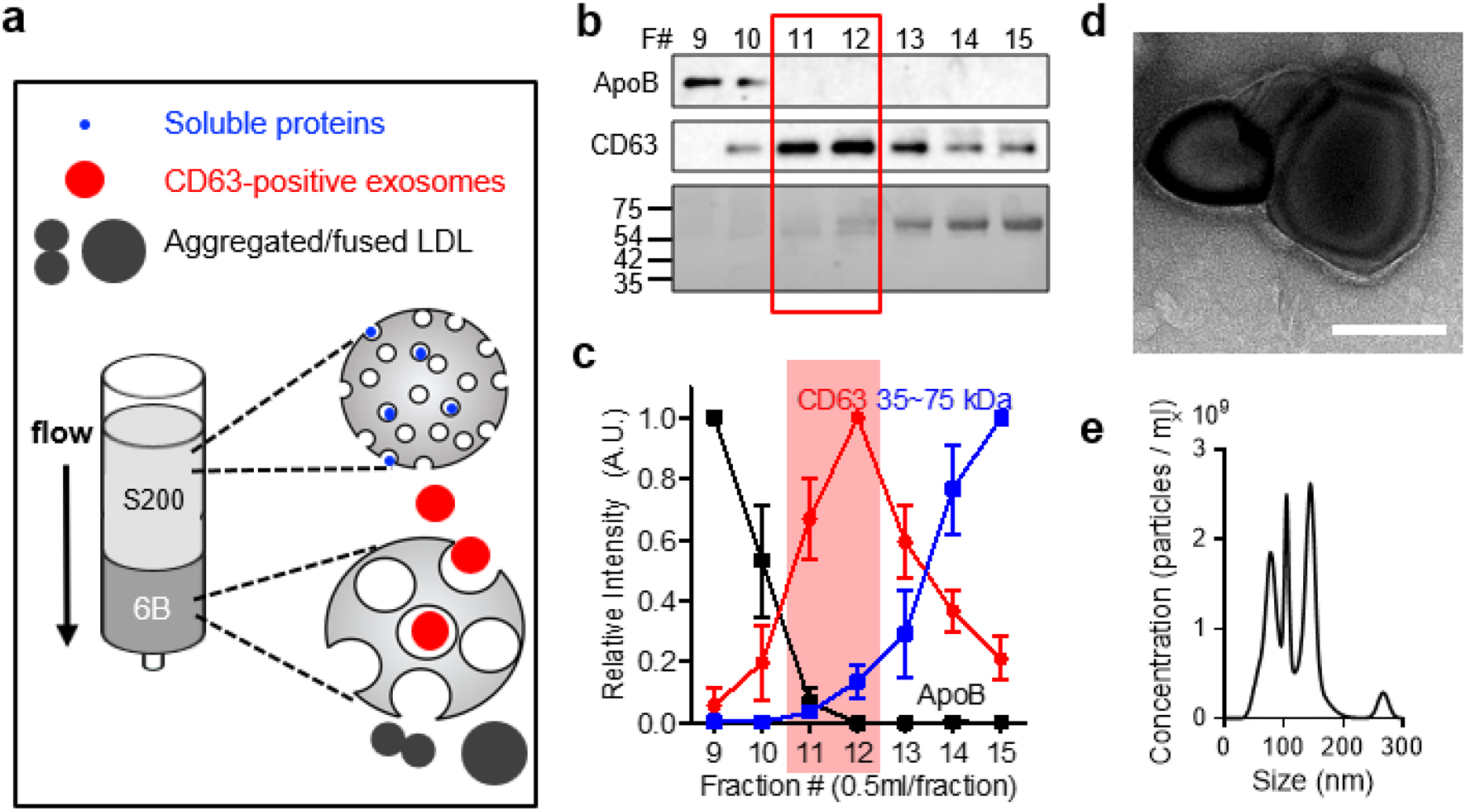
Removal of aggregated LDLs and soluble proteins by dSEC columns. (**a**) Schemetic representation of the developed dual size-exclusion chromatography (dSEC) column. (**b**) Western blot analysis and Ponceau S staining, with equal protein loading, of CD63, ApoB and soluble proteins (MW: 35–75 kDa) upon separation of AML BM plasma by the dSEC column. (**c**) Relative intensities of bands in (**b**) (n = 3). EV-collecting fractions are highlighted in red. (**d**) TEM image of EVs in eluate fractions 11–12. Scale bar: 100 nm. (**e**) Size distribution of the isolated EVs in eluate fractions 11–12.

Even though the CL-6B column was effective in separating EVs from ApoB positive particles, there was still an overlap of soluble protein bands with CD63 bands in the fractions 12–15 where EVs were collected. As such, we designed a dual size-exclusion chromatography (dSEC) column. Sephacryl S-200 h (S-200HR) beads which can interact with soluble proteins with the molecular weight of 5–250 kDa, were stacked on top of CL-6B beads (Fig. 4a). The stacking volume ratio between S-200HR and CL-6B was optimized based on western blot results. The volume ratio which resulted in the highest purity of CD63 was regarded as optimal. When dSEC was filled with 70% of S-200HR beads, relatively purer CD63 bands were detected in fractions 11–12. ApoB bands were intensified in fractions 9–10. (Supplementary Fig. S7). In addition, approximately 17 min were required to collect EVs enriched fractions 11 and 12 (Supplementary Fig. S8). As such, the volume ratio of 70% S-200HR was selected for constructing dSEC column for subsequent experiments. The same elution tendency was not exhibited when the stacking order of the two beads was reversed even though the volume ratio of each bead remained unchanged (Supplementary Fig. S9). The isolation perfomance of dSECs was evaluated by using BM AML plasma samples. Western blotting revealed that ApoB and CD63 bands were intensively detected in fractions 9–10, and fractions 11–13, respectively (Fig. 4c, and Supplementary Fig. S10). Other tetraspanin EVs markers such as CD9 and CD81 were also enriched in fractions 11–12 (Supplementary Fig. S11). A marked increase in the intensity of soluble proteins was visualized from fraction 13 and onwards upon ponceau S staining (Fig. 4c). The elution pattern of soluble proteins was double-confirmed by loading bovine serum albumin (BSA) onto the dSEC column. UV-absorbance measurement revealed a substantial increase in the absorance from fraction 13. In addition, less than 3% of the total protein absorbance was detected in fractions 11 and 12 (Supplementary Fig. S12). This confirmed the efficient removal of soluble proteins by using the developed dSEC column. When EVs in fractions 11–12 were visualized under TEM, the cup-shaped morphology was elucidated (Fig. 4d). The EVs present in fractions 11–12 were having the density of 1.202–1.250 g/ml and the zeta potential value of − 13.6 ± 0.51 mV (Supplementary Fig. S13a,b). In addition, these EVs were having the size range of 40–160 nm when measured by NTA. (Fig. 4e). Taken together, these results suggest that the developed dSEC columns are capable of rapidly isolating EVs from other contaiminats present in human BM plasma.

### Comparison of the performance of the developed dSEC column against conventional isolation methods

**Figure 5:**
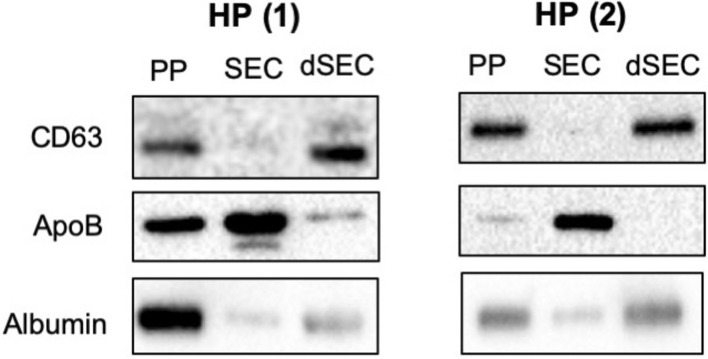
Comparison of the performance of the developed dSEC column and against conventional isolation methods. Western blot analysis of CD63, ApoB and albumin upon isolation of BM plasma (HP) by polymer-based precipitation (PP), conventional SEC (CL-2B), and the dSEC methods.

Lastly, we compared the EV isolation efficiency of the developed dSEC to conventional methods including SEC (CL-2B)^9,10^ and polymer-based precipitation (PP) methods^29,30^. We utilized human BM plasma obtained from two AML patients. Similar CD63 intensity was noted upon utilization of the dSEC when compared to the ones isolated by PP and conventional SEC methods (Fig. 5). In addition, ApoB intensity for the dSEC was significantly lower than the conventional methods. Weak albumin intensity was noted for both dSEC and PP method. These results suggest that the dSEC method is indeed better at isolating EVs with less contaminants when compared to conventional isolation methods.

## Discussion

In this report, we developed a dual size exclusion chromatography column which can rapidly enrich extracellular vesicles (EVs) from BM of AML patients with a single isolation step. It should be noted that no difference in ApoB proportions was observed between BM and peripheral plasma (PB), obtained from the same patient. EV associated markers such as CD9, CD63, and CD81 were intensified in BM samples. As such, BM is considered as the better candidate especially when identifying new EV associated biomarkers for AML (Supplementary Fig. S14).

We utilized conventional CL-2B size exclusion chromatography columns for enriching EVs from BM. However, when the conventional columns are used for enriching EVs from plasma, ApoB positive particles tend to elute at early fractions together with EVs. As such, the contamination of ApoB positive particles has long been a problem with conventional columns when isolating pure EVs^10,12,31,32^ . Moreover, it was even reported that more than 70% of the particles isolated from plasma were in fact, non-extracellular vesicles^10^. There have been several reports which offered possible explanations to the presence of ApoB associated particles in early eluate fractions. First of all, Sodar et al. suggested the possibility of LDL aggregation and binding to EVs, which include microvesicles and exosomes, at physiological conditions^12^. Secondly, Karimi et al. suspected of the size similarity between VLDLs and EVs^16^. However, another cause was proposed in this study. Upon physiochemical analysis of fractions 9–11, ApoB positive particles were mainly detected and these particles exhibited the characteristics of LDLs (density, morphology size, zeta potential, Fig. 2c–e) and not VLDLs. The hydrodynamic sizes of the particles were approximately 100–300 nm (Fig. 2f) implying the likelihood of LDL aggregation. The potential cause for LDL aggregation was confirmed in-vitro and it was attributed to the effect of freezing and thawing. As clinically relevant plasma samples are usually kept under freezing conditions for long-term storage purposes, further research should be performed to elucidate the effect of freezing and thawing on plasma samples.

We evaluated the isolation performance of the dSEC by selecting eluate fractions of high CD63 intensities upon western bloting. CD63 is a tetraspanin protein which is involved in a variety of intracellular processes such as endosomal trafficking and exosome cargo sorting^33,34^. Importantly, the level of CD63 has been reported to be elevated in the plasma of leukemia, melanoma, and colorectal cancer patients^35–37^. In addition, more than 90% of exosomes derived from AML cells are known to express CD63. Other exosomal markers, such as TSG101 are found to be less abundantly expressed^33^.

Recently, new subsets of EVs including non-membranous exomeres, (< 50 nm), small-exosomes (60–80 nm), and large-exosomes (90–120 nm) were discovered. As tetraspanin expressions such as CD9, CD63, and CD81 are known to be enriched in small exosomes, the EVs collected from the dSEC are likely be “small exosomes”^38^. In addition, the zeta potential of EVs isolated by the dSEC (− 13.6 ± 0.51 mV) was similar to the zeta potential of exosomes, about − 9.0 mV to − 12.3 mV. This confirms that the isolated EVs are not exomeres as they have weakly negative charge, in the range between − 2.7 and − 9.7 mV^38^. Futhermore, the EVs (1.202–1.250 g/ml) obtained by the dSEC were falling in the category of high density EVs (HD-Exo, 1.26–1.29 g/ml, less than 100 nm) rather than low density EVs (LD-Exo, 1.12–1.19 g/ml, 70–200 nm) EVs^23^.

CD34 is a known EV associated biomarker that can distinguish between AML patients and health individuals^37,39^. As such, the effectiveness of the newly developed dSEC column was evaluated in terms of its ability in enriching CD34 positive EVs. Upon western blotting, enhanced intensities of CD63 and CD34 bands were noted in fractions 11–12 suggesting the liklihood of CD34 positive EV accumulation in these fractions (Supplementary Fig. S15). At the same time, when plasma from healthy individuals (Supplementary Fig. S16) or PB plasma (Supplementary Fig. S17) was subjected to the dSEC column, high intensity of EV associated markers (CD63 and CD81) were noted in EV collecting fractions. In addition, ApoB bands were found to be dimmed in these fractions. This allows broader application of the developed column, not only limited to BM plasma of AML patients. Even though the absolute number of EVs might be less than the number of EVs originally present in plasma, it should be emphasized that the purity of AML associated EVs isolated was greatly enhanced by utilizing the dual SEC column. This is particularly important for future EV-based proteomics where highly pure EV samples are desired for ultimate identifications of EV associated biomarkers.

## Materials and methods

### Preparation of size exclusion chromatography columns

10 ml of either Sepharose CL-2B (GE Healthcare) or Sepharose CL-6B (GE Healthcare) resin was utilized to construct a single size exclusion chromatography column (SEC). A dual size exclusion chromatography column (dSEC) was prepared by stacking predetermined volumes of Sepharose CL-6B and Sephacryl 200-h (Swiss, GE Healthcare) resins. The constructed columns were then vigorously washed with 30 ml of Dulbecco’s phosphate buffered saline (DPBS, Welgene) and stored at 4 °C until usage.

### Cell culture

THP-1 (ATCC#TIB-202), HL-60, human Mesenchymal stem cell (hMSC), and human Dermal Fibroblasts (HDF) cells were cultured at 37 °C and 5% CO_2_ in RPMI 1640 or Iscove's Modified Dulbecco's Medium supplemented with 10% exosome-depleted fetal bovine serum (dFBS), and 1% penicillin and streptomycin. When approximately 50% confluency (~ 2.0 × 10^5^–5.0 × 10^5^ cells/ml) was reached, supernatants were collected for further investigations.

### Sample preparation from cell culture media

Samples prepared from cell culture media were subjected to sequential centrifugations in prior to size exclusion chromatography. Briefly, supernatants of cell culture media were differentially centrifuged at 500×g, for 10 min and 5000×g, for 30 min at 4 °C to eliminate cells, cell debris, and apoptotic bodies, followed by ultracentrifugation at 10,000×g for 30 min at 4 °C for removing microvesicles. A 50 ml of differentially centrifuged product was concentrated 100 times by using Amicon ultra 100 kDa filters (Merck Millipore) and was loaded on top of the size exclusion chromatography column filled with 10 ml of Sepharose CL-2B (GE healthcare Life Sciences) resin. Individual fractions of 0.5 ml eluate were collected and concentrated using Amicon ultra 100 kDa filters (Merck Millipore) for further analysis.

### Sample preparation from human plasma

All human samples were provided by the Division of Hematology-Oncology and the Department of Internal Medicine, Korea University College of Medicine. Sample preparations were performed as per guidelines of the Institutional-Review-Board of Korea University (Anam), Guro Hospital (approved protocol Nos. 2015AN0267 and 2014GR0089) and Korea Advanced Institute of Science and Technology (KH2019-12). All studies were performed in accordance with the principles of the Declaration of Helsinki. Informed consents were obtained from all participants. Only the subjects with more than 18 years of age were selected in this study. After centrifuging human plasma samples at 10,000×g, 4 °C for 30 min, supernatants were collected and loaded on top of SEC or dSECs (Sepharose CL-6B/Sephacryl 200-h = 3/7). Afterwards, individual fractions of 0.5 ml eluate were collected. In parallel with size exclusion chromatography, a polymer-based exosome precipitation (Invitrogen cat# 4484450) and an anti-CD63 magnetic bead-based exosome isolation (Invitrogen cat# 10606D) methods were performed following manufacturers’ instructions.

### Quantifications of proteins

Bicinchoninic acid assay (BCA) was used according to manufacturer’s instructions (Pierce, Thermo scientific) for protein quantification. Firstly, 5 ul of EV samples were lysed with 5 ul of 0.1% TritonX-100 (Sigma-Aldrich). 200 μl of mixed BCA reagents of A and B, was added to the lysed EV samples. Afterwards, the samples were incubated at 37 °C for 30 min and the absorbance of each sample was measured at 562 nm. For determining the elution pattern of albumins from size exclusion chromatography, a volume of 5.0 g/dL of albumin solution was loaded on top of a size exclusion column and the resulting eluate fractions were subjected to absorbance measurements at 280 nm by utilizing an UV–Vis spectrophotometer (SpectraMax Plus384, Molecular Device).

### Nanoparticle tracking analysis

The size distribution of particles in the sample was determined by nanoparticle tracking analysis (NTA). The 10–100 diluted samples were suspended in PBS and determined the size repeated 3 times using Nanosight instrument (Malvern Instruments, Malvern, UK) at 25 °C.

### Dynamic light scattering

Size distributions and zeta potentials of EV samples were measured by using dynamic light scattering (DLS) and zetasizer (Zetasizer Nano ZS90, Malvern Instrument), equipped with a solid-state laser adjusted at 633 nm. All eluates were measured without dilution while commercial LDLs were measured after a tenfold dilution.

### Transmission electron microscopy (TEM)

Isolated EV fractions were mixed with an equal volume of 4% paraformaldehyde (PFA). For visualizing low density lipoproteins (Fitzgerald, USA), samples were diluted by 100 folds. 10 μl of EV or LDL sample solutions were overlaid with Formvar-carbon coated electron-microscope grids and incubated for 15 min. Next, the grids were washed with DPBS and fixed for 5 min with 2.5% glutaraldehyde. Afterwards, the grids were washed with distilled water for five times and incubated in phosphotungstic acid (pH 7) for 3 min. Excess stain was removed by blotting with a filter paper. The dried grids were then imaged with a transmission electron microscope at 200 kV.

### Ponceau S and western blot

Protein concentrations of individual eluate fractions obtained from 3 different plasma samples were measured by bicinchoninic acid assay (BCA) according to manufacturer’s instructions. Samples were then either concentrated or diluted to have a concentration value within the range of 300 µg/ml and 2000 µg/ml. Equal protein amount of each sample was mixed with reducing 5 × Laemmli sample buffer (Elpis-Biotech) and heated for 3 min at 95 °C. Proteins were then separated by sodium dodecyl sulfate–polyacrylamide gel electrophoresis (SDS-PAGE) by using 4–20% gradient polyacrylamide gels (Bio-Rad, Cat #: 456-1093). Membranes were transferred to a nitrocellulose membrane by using Trans-Blot Turbo system (Bio-Rad). Afterwards, the nitrocellulose membranes were stained with *Ponceau S* solution for visualizing protein bands by using ChemiDoc imaging system (Bio-Rad). For subsequent immunostaining, the membranes were first blocked with 5 w/v% skim milk in Tris-buffered saline supplemented with 0.1% Tween-20 (TBST) for an hour and incubated with the following antibodies overnight at 4 °C: anti-ApoB (1:2000, Santa Cruz Biotechnology, sc-25542), anti-CD9 (1:1000, Santa Cruz Biotechnology, sc-9148), anti-CD63 (1:1000, Bioss, bs-1523R), anti-CD81 (1:1000, Bioss, bs-6934R), and anti-albumin (1:8000, Bioss, bs-0945R). After 5 washes with TBST, the membranes were incubated with horseradish peroxidase conjugated anti-rabbit secondary antibodies for 2 h at room temperature (1:2000, Cell Signaling Technology, 7074S). The membranes were then washed 5 times with TBST and incubated with Clarity western ECL buffer (Bio-Rad, Cat #: 170-5060) for 15 min. Finally, the immunoreactive bands were visualized by ChemiDoc imaging system (Bio-Rad). Intensities of bands were analyzed by using Image Lab Software 5.2 (Bio-Rad).

For data analysis, the intensities of protein bands were compared with the band intensity with the highest value. Specifically, the band with the highest intensity was set to 1. Western blot intensities were not normalized by the Ponceau S staining to avoid staining bias, which might occur upon loading insufficient amount of proteins (Supplementary Fig. S19a). To ensure the controlled loading, Western blot results were normalized against Ponceau S staining only in case that the sufficient amount of proteins were present. No significant difference in elution trends was noted when the normalized Western blot results were compared with its unnormalized counterpart (Supplementary Fig. S19b). Although the performed analysis method was confirmed that there is no effect in elution trends of Western blot, there remains a limitation in that only approximate overall purity can be confirmed.

### Iodixanol density gradient ultracentrifugation

Iodixanol density gradient ultracentrifugations were performed according to the previous protocol with minor modifications^15^. OptiPrep containing 60 w/v % of iodixanol was utilized in this study. Human plasma samples and eluate fractions of SEC and dSECs were loaded onto 0–60% iodixanol gradient layers and placed in a SW41 swinging bucket (Beckman) for ultracentrifuge at 200,000×g, 4 °C, 16 h. Total 12 fractions of density gradient layers were collected after centrifugation. The mass of 100 μl, obtained from each fraction was measured (Satorius, CPASS4S) and its corresponding density was calculated. Afterwards, the correlation between iodixanol concentration and density was utilized to determine the unknown density of a particular sample.

### Statistical analysis

All data were presented as mean ± SD from technical replicates, unless otherwise indicated. The significance between groups was measured using the Student’s t-test or one-way analysis of variance followed by the Tukey’s multiple comparison test. Statistical testing was performed using GraphPad Prism 7.0 (GraphPad software). *P* values < 0.05 were considered statistically significant (*P < 0.05, **P < 0.01 and ***P < 0.001).

## Supplementary information

Supplementary Information
