# Supplementary material for: Dual size-exclusion chromatography for efficient isolation of extracellular vesicles from bone marrow derived human plasma
Source: Sci Rep. 2021 Jan 12;11:217. doi: 10.1038/s41598-020-80514-8 (PMC7804108; doi:10.1038/s41598-020-80514-8)
Supplement: Supplementary file 1 — Supplementary Information [file 41598_2020_80514_MOESM1_ESM.pdf]

Supporting Information for:

Dual size-exclusion chromatography for efficient isolation of extracellular vesicles from bone marrow derived human plasma

Jik-Han Jung[1], Woojin Back[1], Junyong Yoon[1], Hyeonjeong Han [1], Ka-Won Kang[2], Byeonghyeon Choi[3], Hyesun Jeong[4], Jaena Park[5], Hyunku Shin[5], Woojune Hur[2], Yeonho Choi[5], Sunghoi Hong[4], Hyun Koo Kim[3], Yong Park[2], and Ji-Ho Park[1,*]

[1]Department of Bio and Brain Engineering and KAIST Institute for Health Science and Technology, Korea Advanced Institute of Science and Technology (KAIST), Daejeon, Republic of Korea

[2]Division of Hematology-Oncology, Department of Internal Medicine, Korea University College of Medicine, Seoul, Republic of Korea

[3]Department of Thoracic and Cardiovascular Surgery and Department of Biomedical Sciences, Korea University College of Medicine, Seoul, Republic of Korea

[4]School of Biosystems and Biomedical Sciences, Korea University, Seoul, Republic of Korea

[5]Department of Biomedical Engineering, Korea University, Seoul, Republic of Korea

*Email: jihopark@kaist.ac.kr

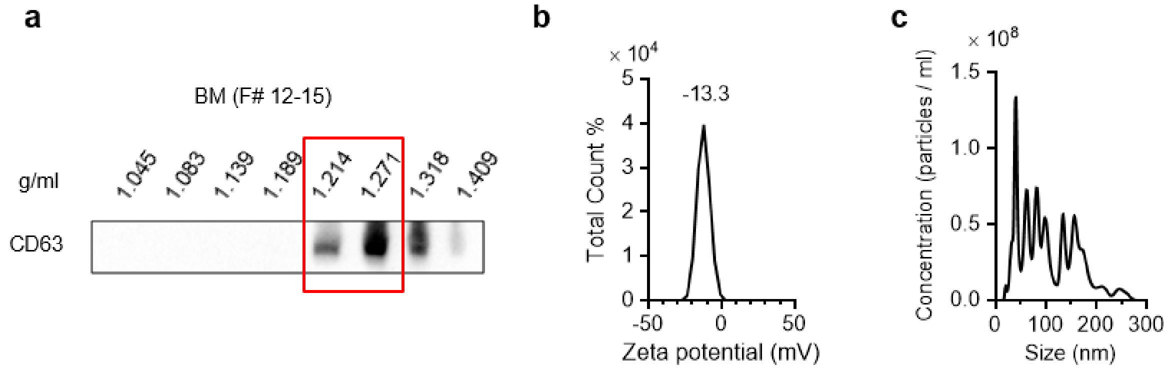

Supplementary Figure S1. Characterizations of the isolated AML BM derived EVs found in eluate fractions 12-15 of CL-2B column. (a) Western blot analysis on eluate fractions 12-15 of AML BM plasma after density gradient ultracentrifugation. Red box indicates the density of the isolated EVs. (b) Zeta potential of the isolated EVs. (c) Size distribution of the isolated EVs.

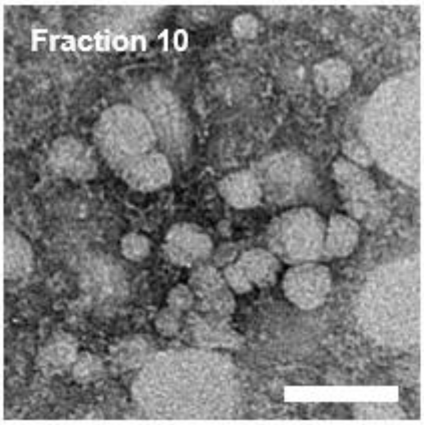

Supplementary Figure S2. TEM image of the particles present in eluate fraction 10 of BM plasma. Scale bar: 100nm.

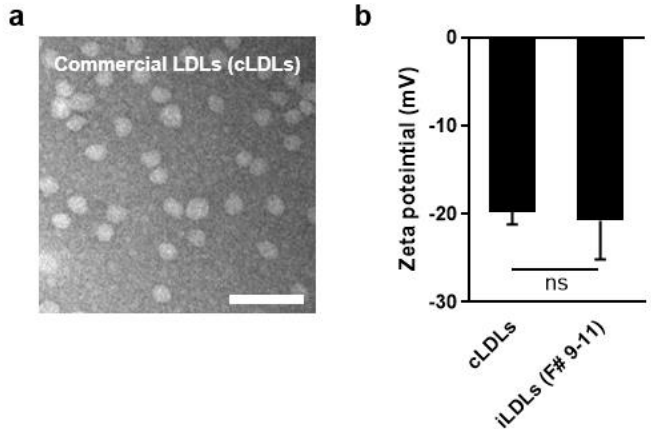

Supplementary Figure S3. (a) The morphology of commercial LDLs, visualized under TEM. Scale bar: 100 nm. (b) Comparison between the zeta potentials of the isolated LDLs (iLDLs) and commercial LDLs (n = 5, by Student *t*-test)

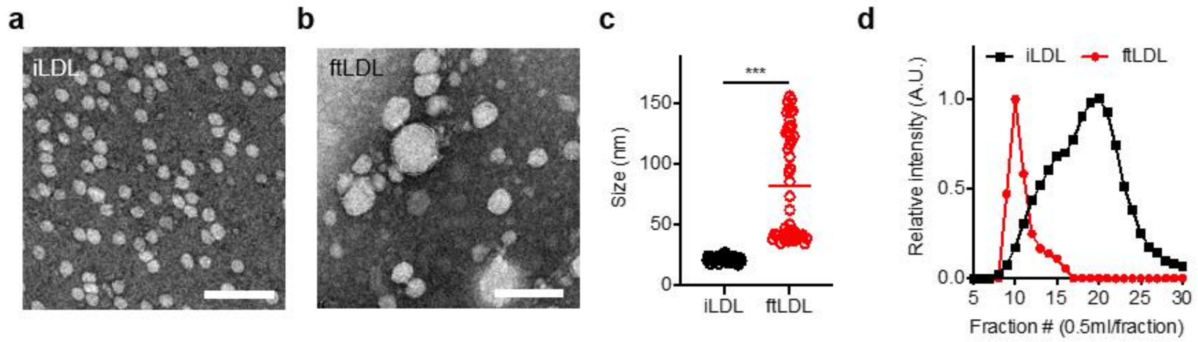

Supplementary Figure S4. The effect of freezing and thawing on LDL aggregation in vitro. (a and b) TEM images of iLDLs and ftLDLs (c) Size distributions of iLDLs and ftLDLs (n = 51; ***P < 0.001 by Student *t*-test). (d) Elution tendencies of iLDLs and ftLDLs from CL-2B column. (n = 3, by Student *t*-test). Scale bar: 100nm.

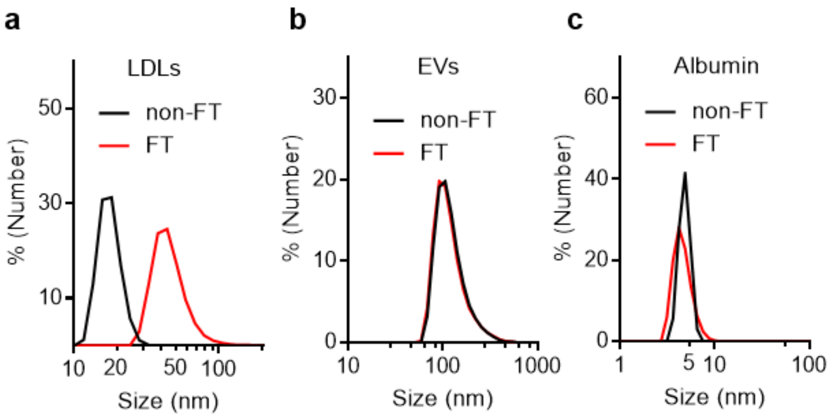

Supplementary Figure S5. The effect of freezing and thawing on the size of LDLs, EVs and albumins. (a-c) Hydrodynamic sizes of commercial LDLs, THP-1 cell culture medium-derived EVs, and albumins upon freezing and thawing.

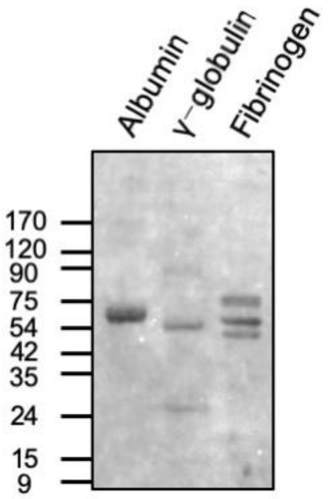

Supplementary Figure S6. Visualizations of albumins, γ-globulins, and fibrinogens in plasma by Ponceau S staining.

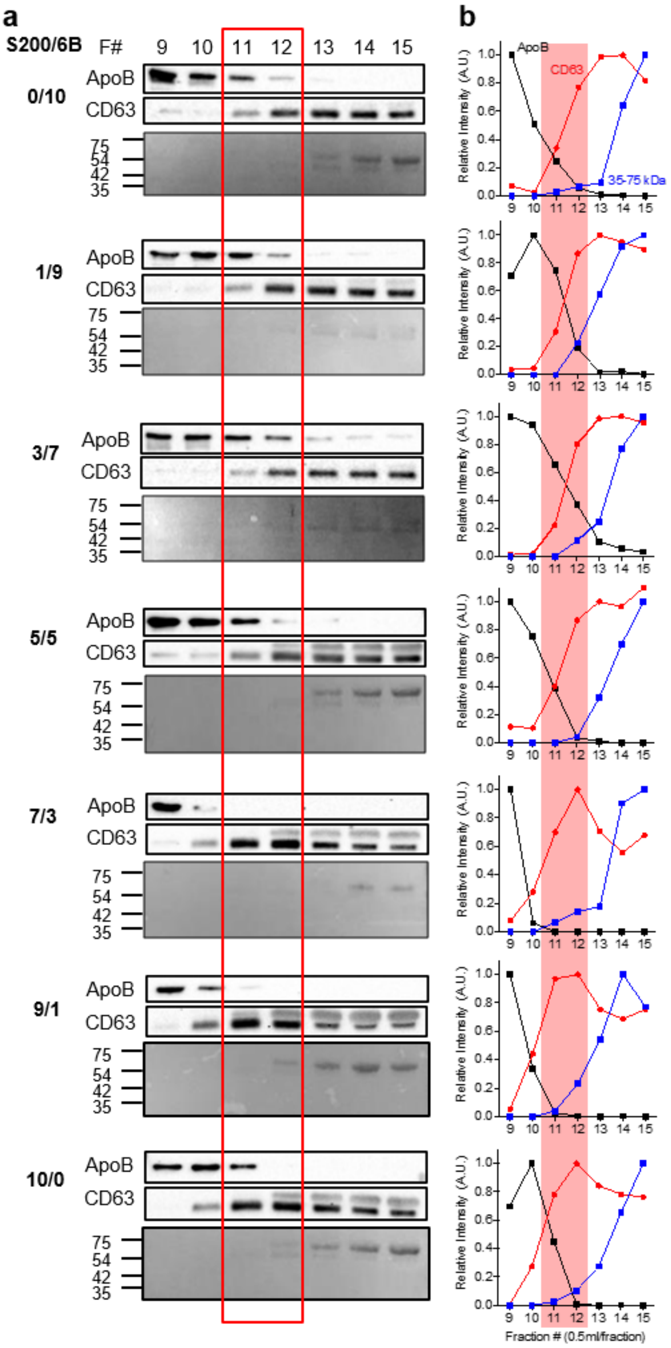

Supplementary Figure S7. Optimization of the S200/6B volume ratio for efficient separation of EVs from LDLs and soluble proteins. (a) Western blot analysis and Ponceau S staining, with equal protein loading, of CD63, ApoB and soluble proteins (MW: 35-75 kDa) upon separation of AML BM plasma by the dSEC column. (b) Relative intensities of protein bands in (a). EV-collecting fractions are highlighted in red.

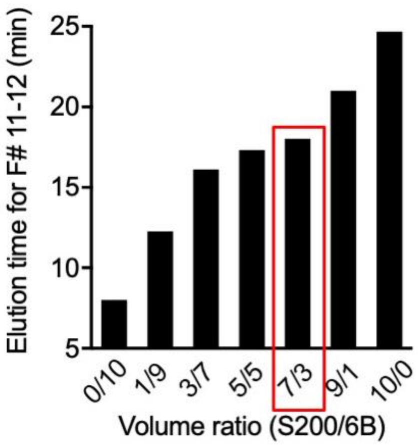

Supplementary Figure S8. Time taken to collect fractions 11-12 with the dSEC of varying S200/6B volume ratios. The selected dSEC ratio are highlighted in red.

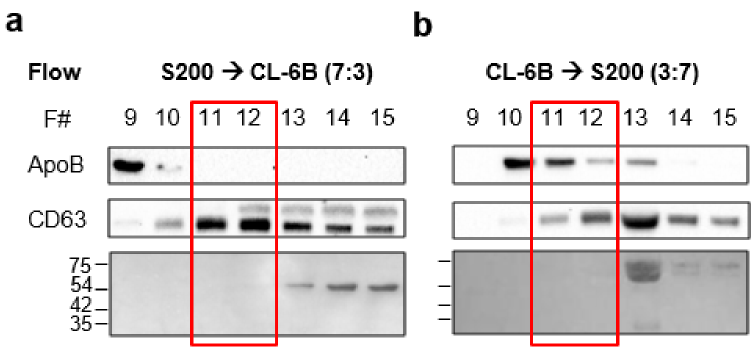

Supplementary Figure S9. Reduction in the isolation efficiency of EVs upon reversal of stacking volume ratios. (a) Western blot analysis and Ponceau S staining, with equal protein loading, of CD63, ApoB and soluble proteins (MW: 35-75 kDa) upon separation of AML BM plasma by the dSEC column. (b) Relative intensities of protein bands in (a). EV-collecting fractions are highlighted in red.

.

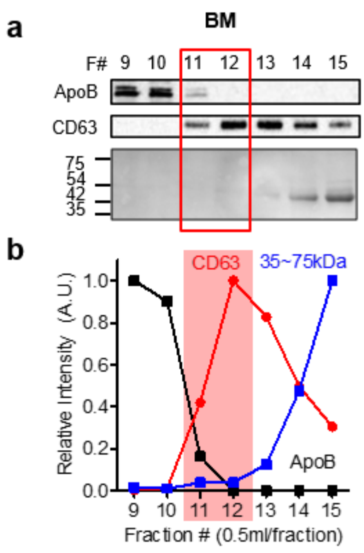

Supplementary Figure S10. Enhancement in the isolation efficiency of EVs at S-200 HR/CL-6B volume ratio of 7 to 3. (a) Western blot analysis and Ponceau S staining, with equal protein loading, of CD63, ApoB and soluble proteins (MW: 35-75 kDa) upon separation of AML BM plasma by the dSEC column. (b) Relative intensities of protein bands in (a). EV-collecting fractions are highlighted in red.

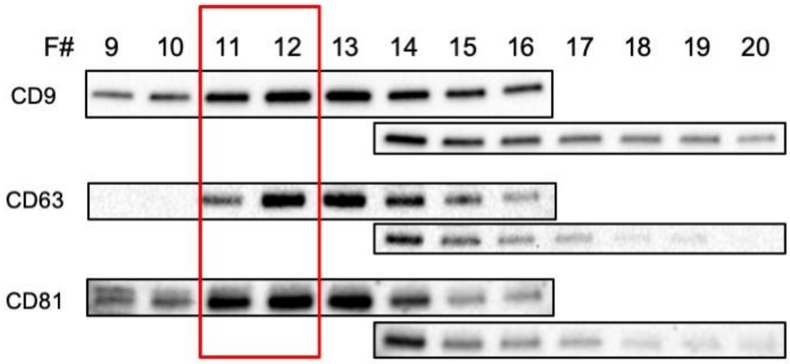

Supplementary Figure S11. Presence of tetraspanin EV markers in eluate fractions 11-12. Western blot analysis of CD63, CD9, and CD81 upon separation of AML BM plasma by the dSEC column. EV-collecting fractions are highlighted in red

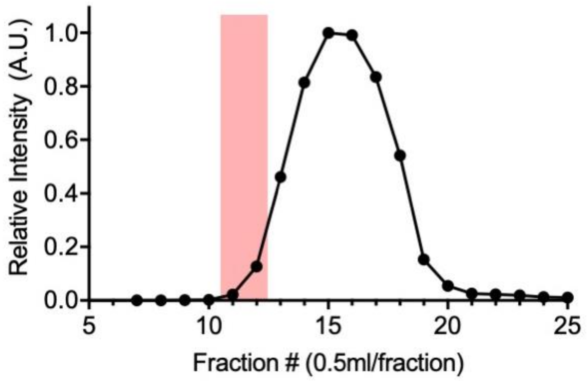

Supplementary Figure S12. Elution tendency of bovine serum albumin (BSA) by the dSEC column. Enrichment of EVs in eluate fractions 11-12.

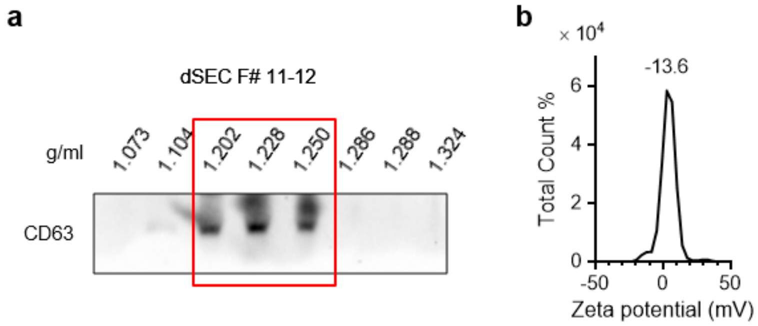

Supplementary Figure S13. **Characterizations of the isolated EVs in fraction 11-12 of AML BM plasma separated by the dSEC column.** (a) Western blot analysis of CD63 after density gradient ultracentrifugation on dSEC fractions 11-12 of AML BM plasma. (b) Zeta potential of the isolated EVs in (a). EV-collecting fractions are highlighted in red.

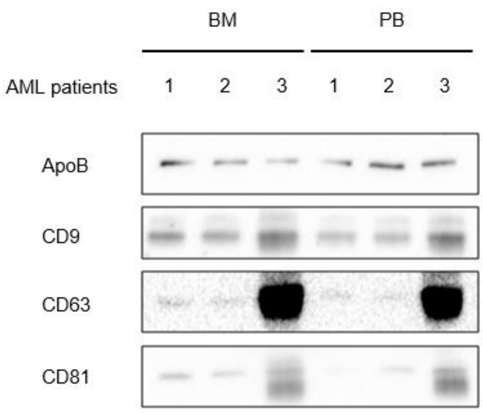

Supplementary Figure S14. Comparison of protein contents in BM and PB plasma, obtained from the same AML patient (n=3).

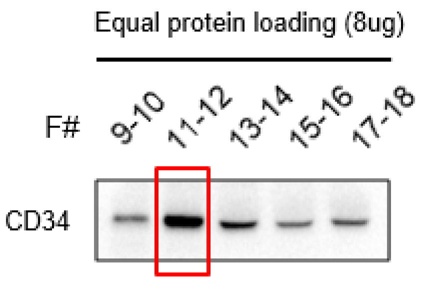

Supplementary Figure S15. Enrichment of CD34 in eluate fractions 11-12 upon separation of AML BM plasma by the dSEC. EV-collecting fractions are highlighted in red

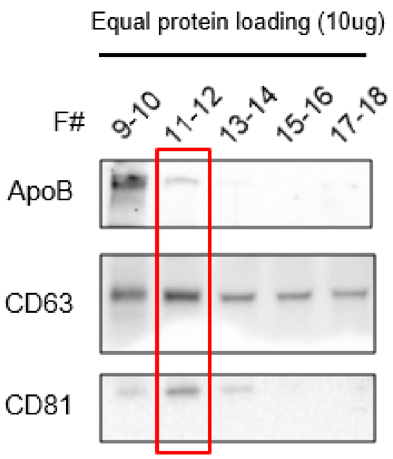

Supplementary Figure S16. Expressions of ApoB, CD63, and CD81 in eluate fractions of BM plasma when separated by the dSEC. EV-collecting fractions are highlighted in red

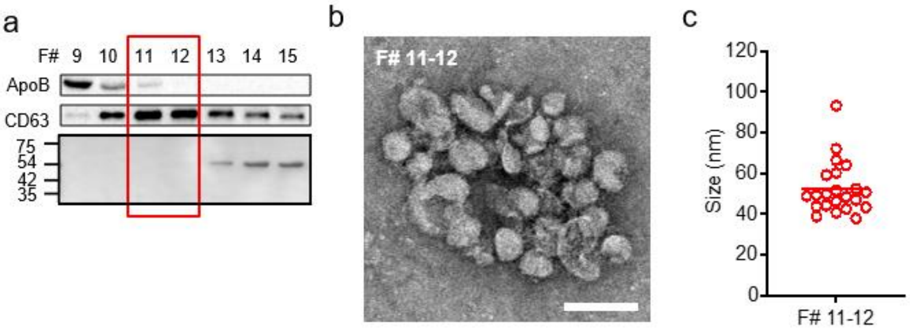

Supplementary Figure S17. Dual size-exclusion chromatography is effective for enriching high-purity EVs from PB plasma of non-small cell lung cancer patients. (a) Western blot analysis and Ponceau S staining, with equal protein loading, of CD63, ApoB and soluble proteins (MW: 35-75 kDa) upon separation of AML BM plasma by the dSEC column. Red box indicates the EV-collecting fractions. (b) TEM image of EVs found in eluate fractions 11-12. Scale bar: 100nm. (c) Size distribution of EVs in eluate fractions 11-12.

Supplementary Figure S18. Un-cropped scans of original western blots used in the main and supplementary figures. Red dotted box indicates the cropped area used in this study

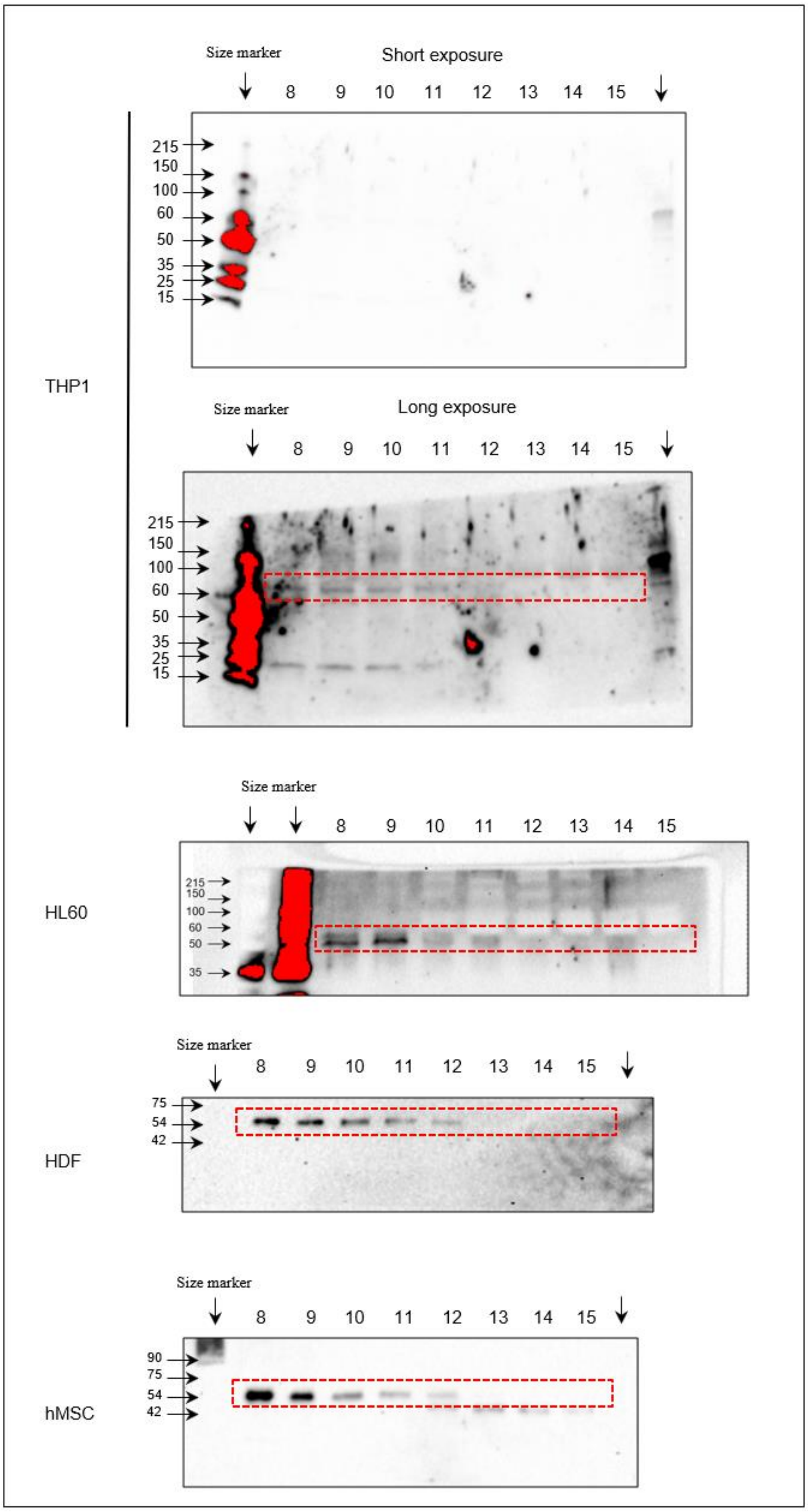

Blots for Fig.1a

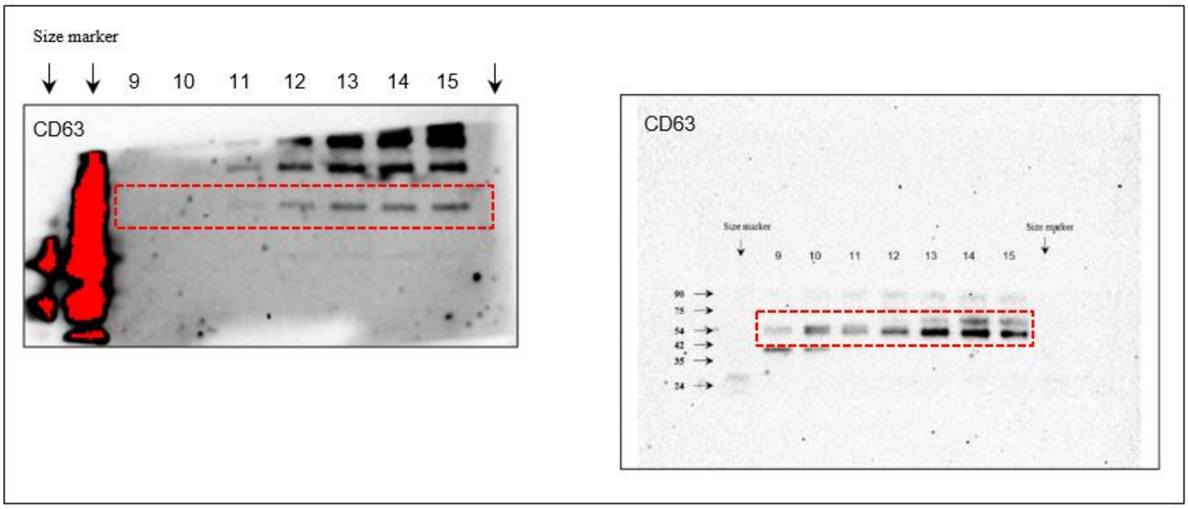

Blots for Fig.1e

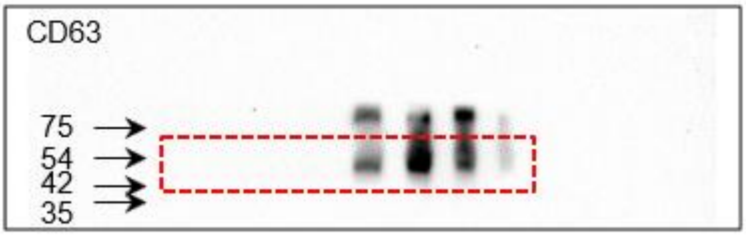

Blots for Supplementary Fig. S1

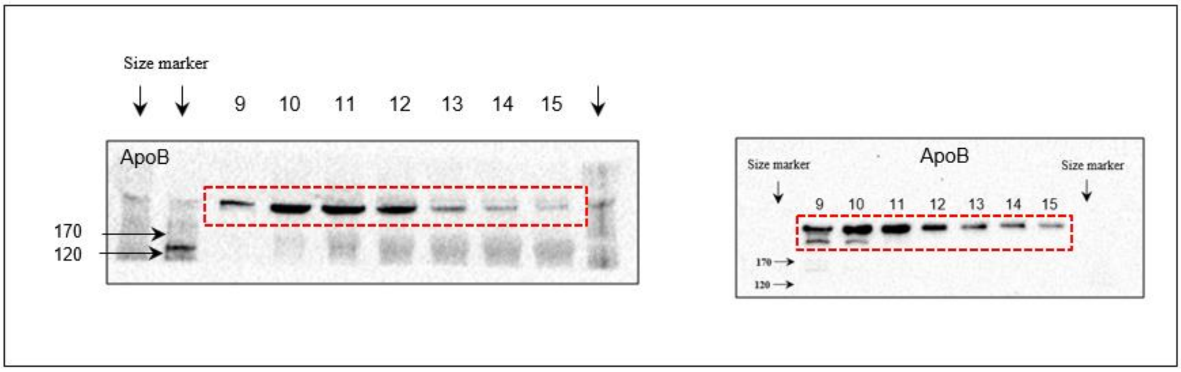

Blots for Fig. 2b

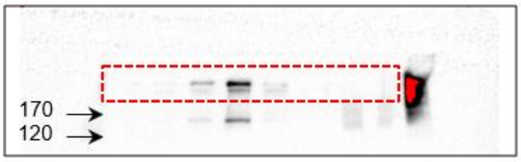

Blots for Fig. 2c

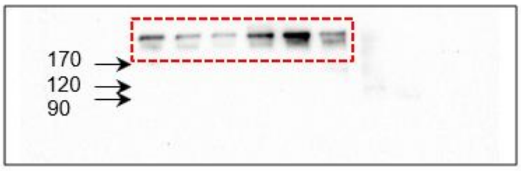

Blots for Fig. 2g

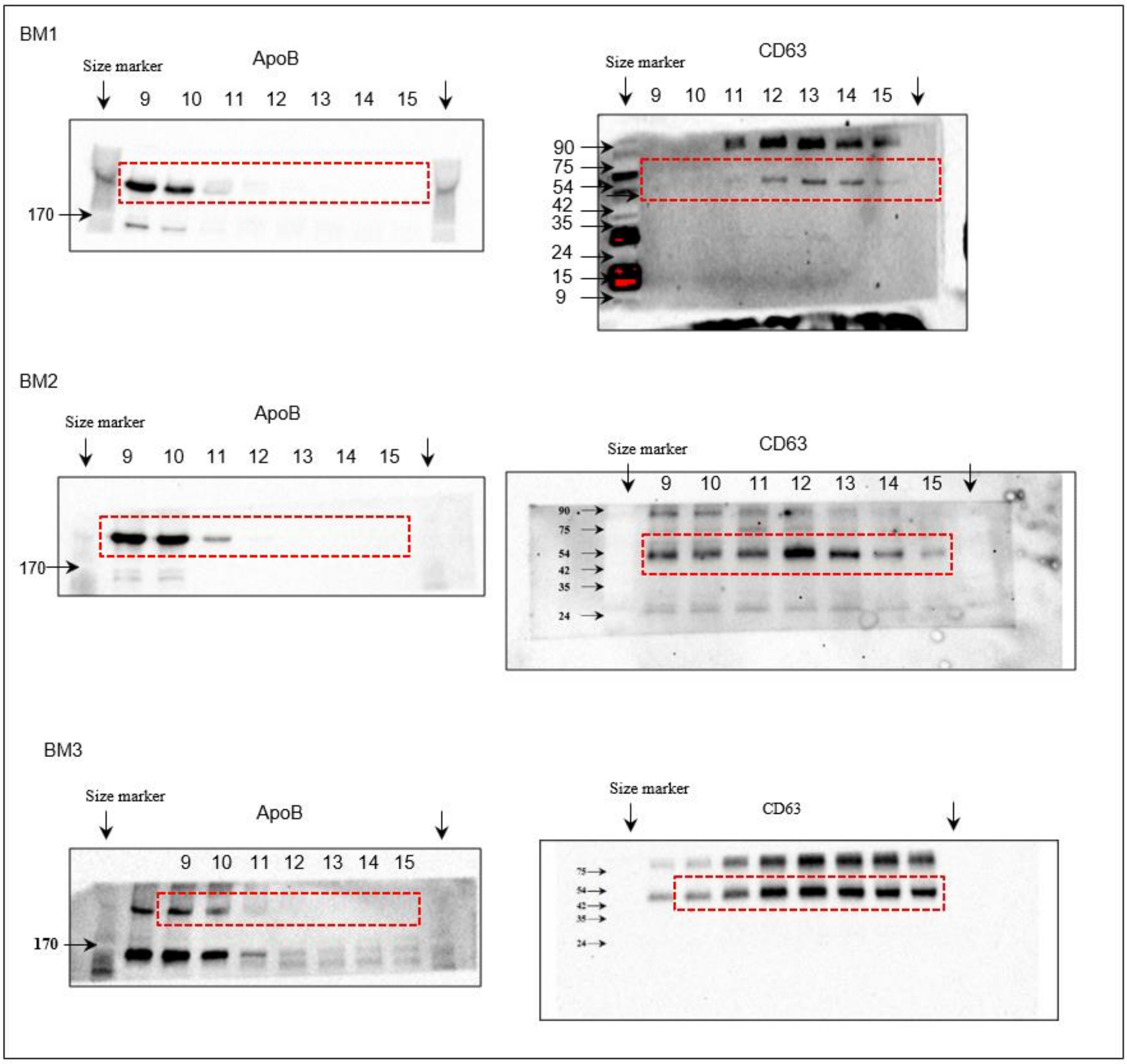

Blots for Fig. 3

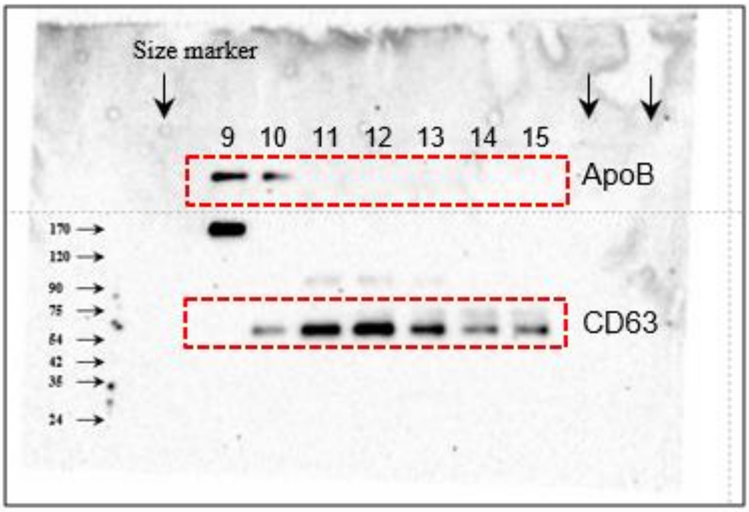

Blots for Fig. 4b and c

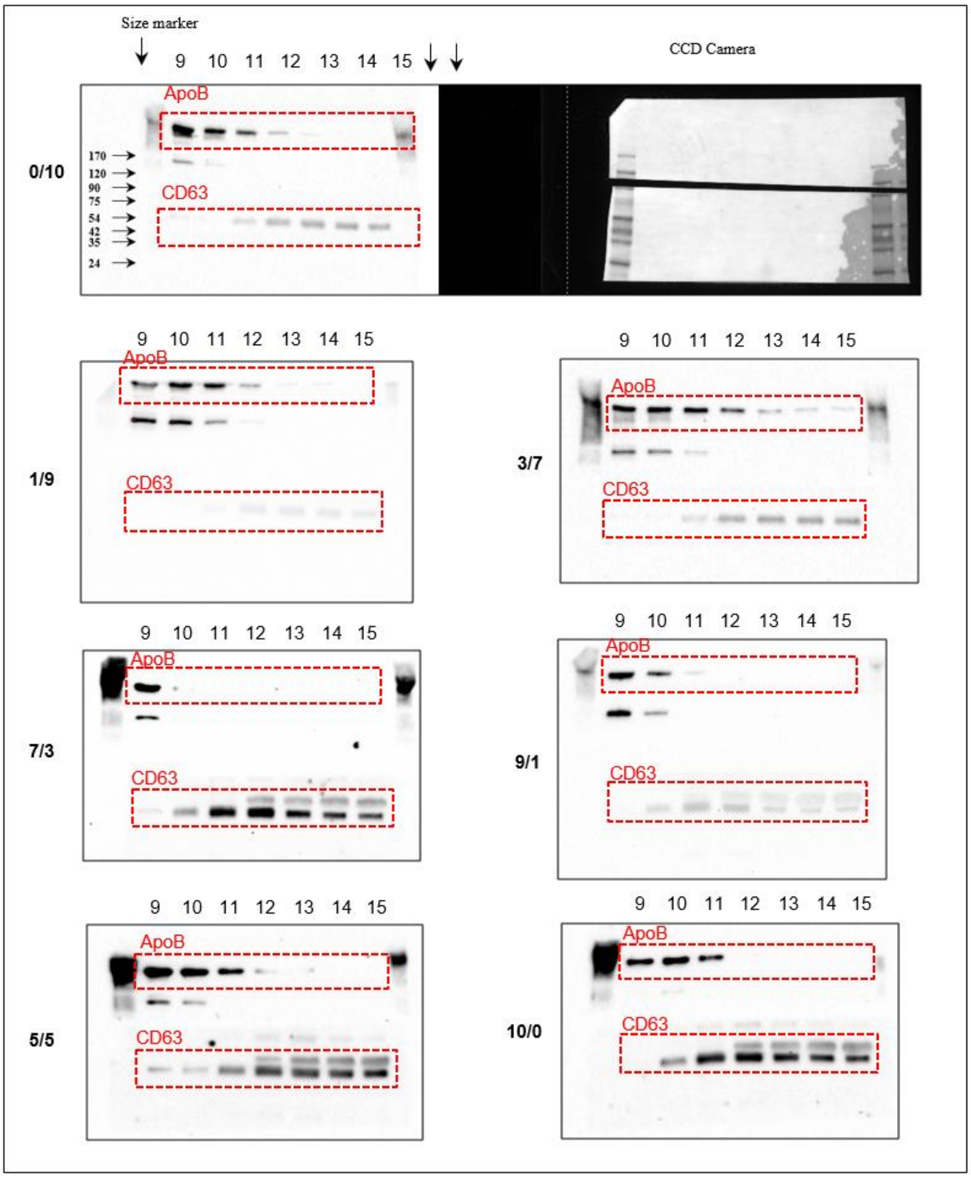

Blots for Fig. 4c & Supplementary Fig. S7

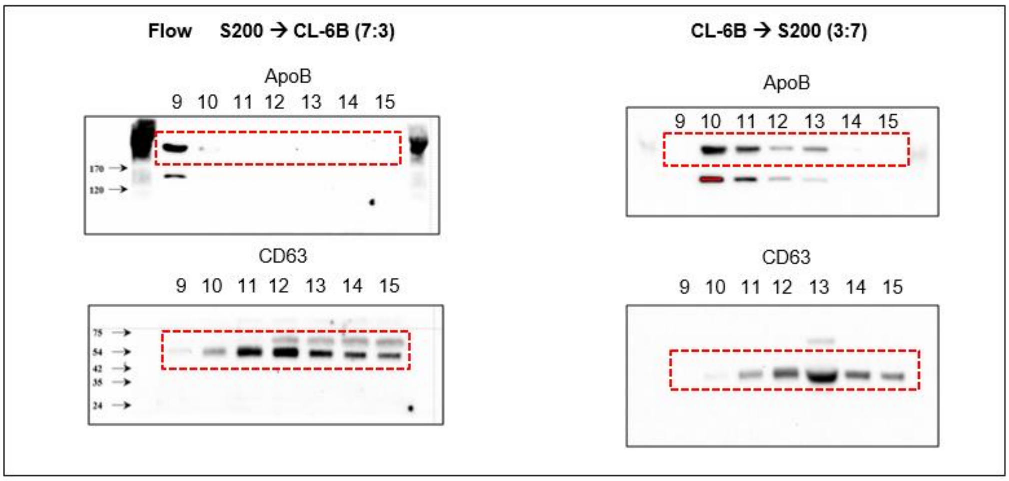

Blots for Supplementary Fig. S9

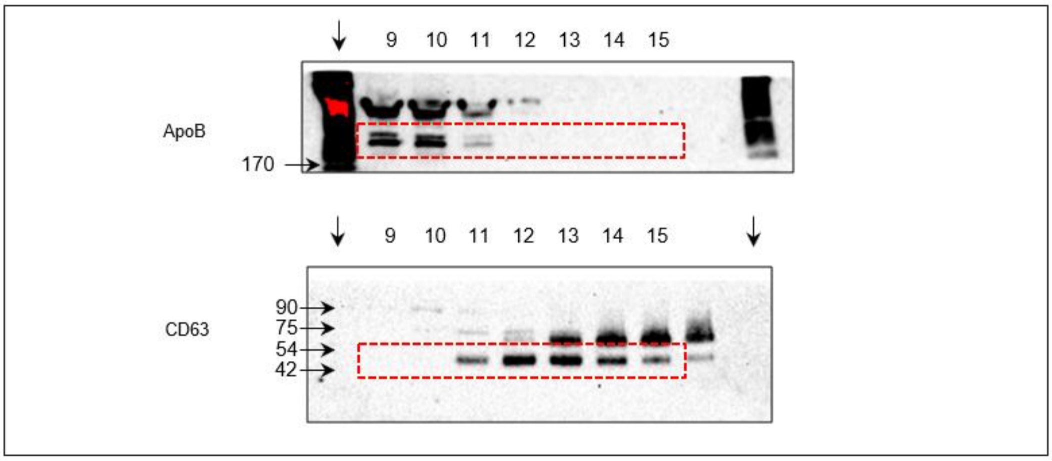

Blots for Supplementary Fig. S10

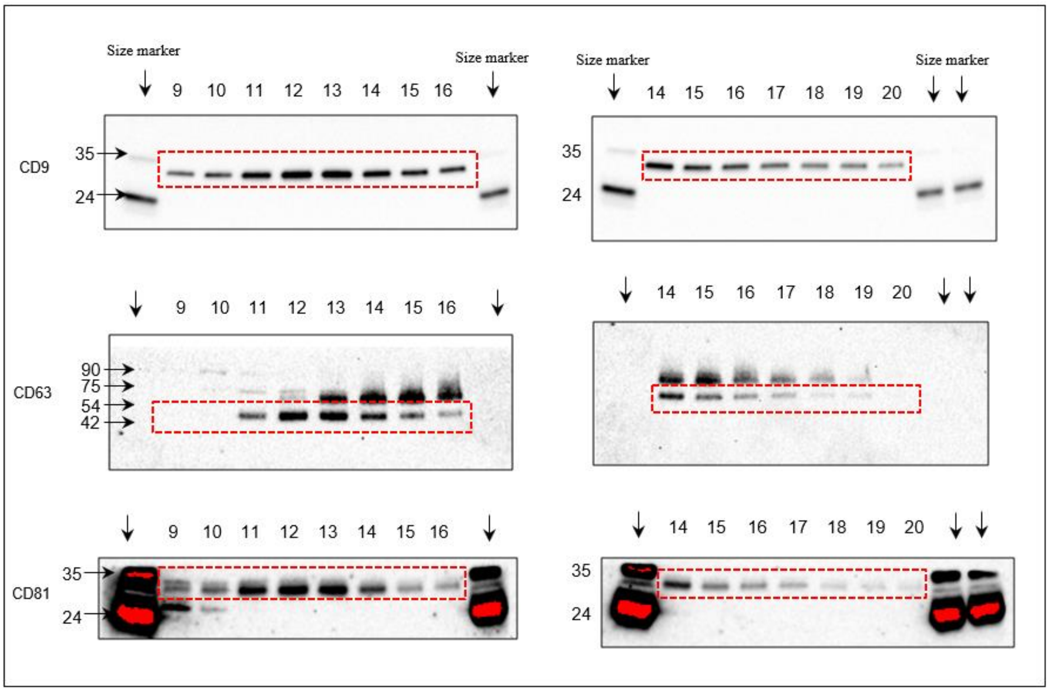

Blots for Supplementary Fig. S10

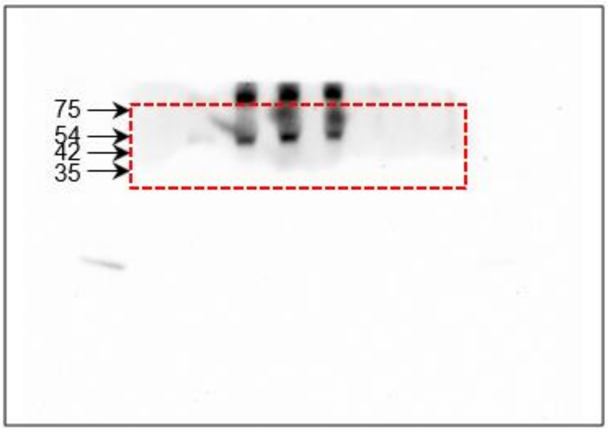

Blots for Supplementary Fig. S13

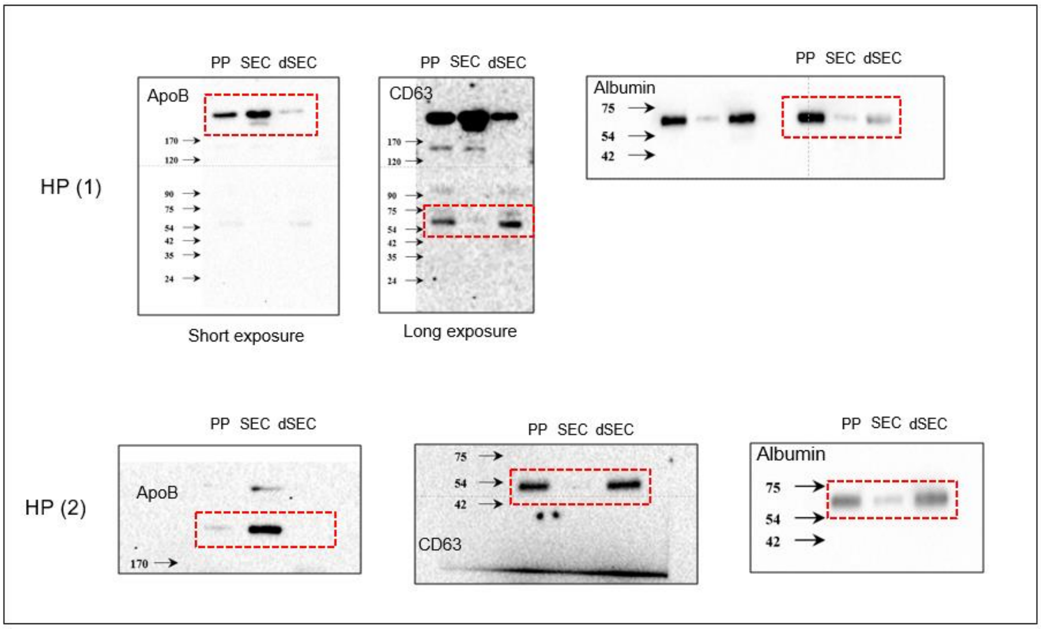

Blots for figure 5

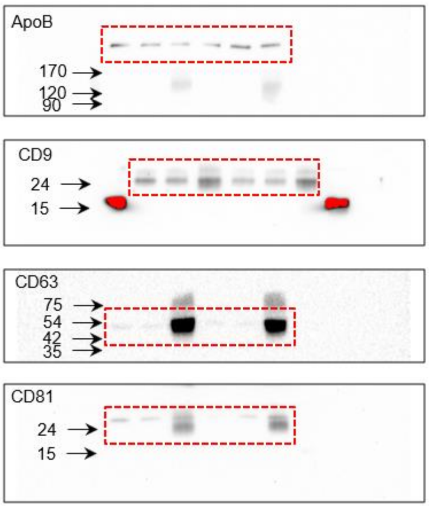

Blots for Supplementary Fig. S14

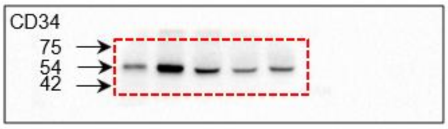

Blots for Supplementary Fig. S15

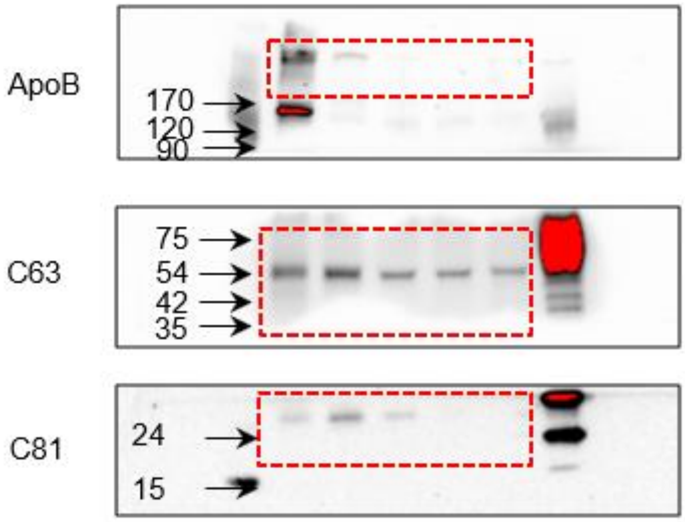

Blots for Supplementary Fig. S16

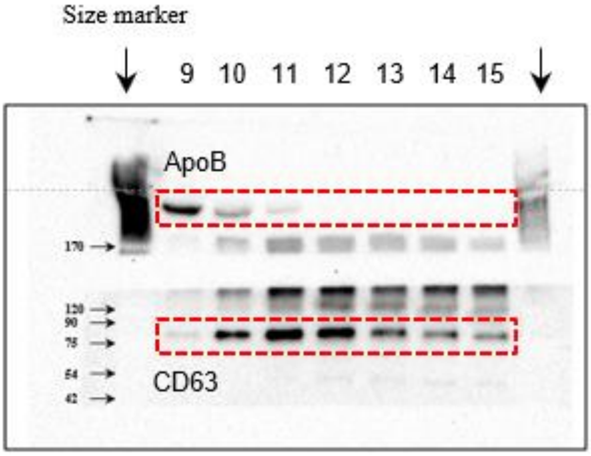

Blots for Supplementary Fig. S17

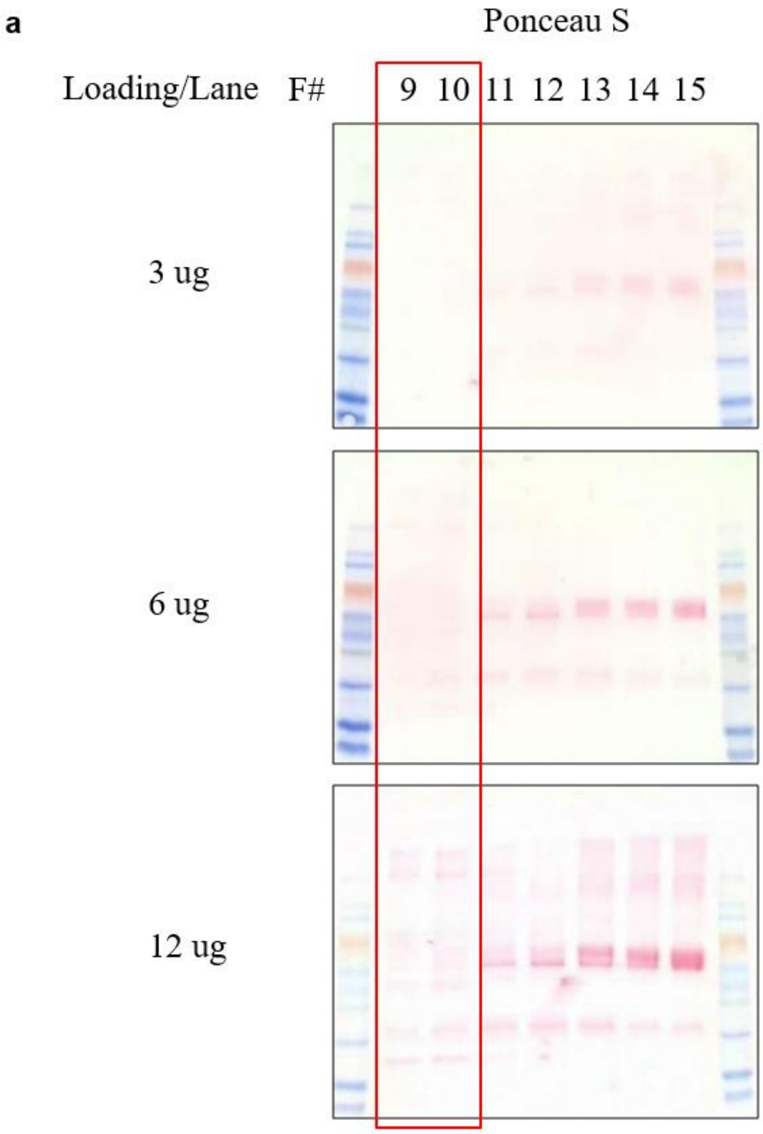

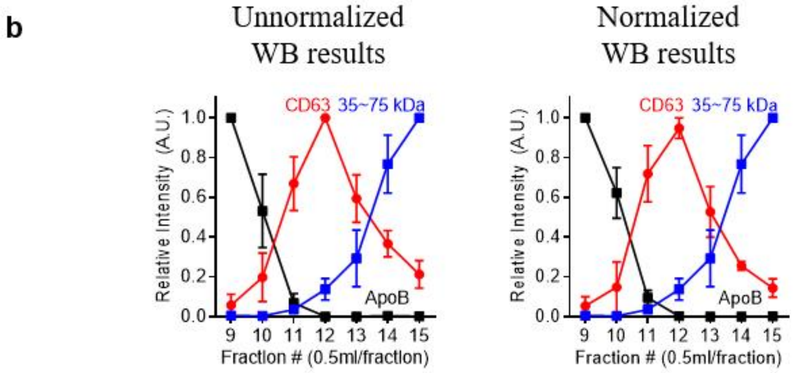

Supplementary Figure S19. (a) The staining bias of Ponceau S (red box) upon loading insufficient amount of proteins. (b) Comparison between the unnormalized and normalized Western blot results.
